# Mechanical Signature of Red Blood Cells Flowing Out of a Microfluidic Constriction Is Impacted by Membrane Elasticity, Cell Surface-to-Volume Ratio and Diseases

**DOI:** 10.3389/fphys.2020.00576

**Published:** 2020-06-12

**Authors:** Magalie Faivre, Céline Renoux, Amel Bessaa, Lydie Da Costa, Philippe Joly, Alexandra Gauthier, Philippe Connes

**Affiliations:** ^1^Université de Lyon, Institut des Nanotechnologies de Lyon INL-UMR 5270 CNRS, Université Lyon 1, Villeurbanne, France; ^2^Laboratoire Interuniversitaire de Biologie de la Motricité (LIBM) EA7424, Equipe “Biologie Vasculaire et du Globule Rouge”, UCBL1, Villeurbanne, France; ^3^Laboratoire d’Excellence (Labex) GR-Ex, Paris, France; ^4^Biochimie des Pathologies Érythrocytaires, Centre de Biologie et de Pathologie Est, HCL, Bron, France; ^5^AP-HP, Service d’Hématologie Biologique, Hôpital Robert-Debré, Paris, France; ^6^Université Paris Diderot, Université Sorbonne, Paris Cité, Paris, France; ^7^INSERM U1149, CRI, Faculté de Médecine Bichat-Claude Bernard, Paris, France; ^8^Institut d’Hématologie et d’Oncologie Pédiatrique (IHOP), Hospices Civils de Lyon, Lyon, France; ^9^Institut Universitaire de France, Paris, France

**Keywords:** mechanical phenotype, microfluidics, red blood cells, pathologies, chemical treatment

## Abstract

Despite the fact that Red Blood Cells (RBCs) have been intensively studied in the past 50 years to characterize mechanical phenotypes associated with both healthy and pathological states, only ektacytometry (i.e., laser diffractometry) is currently used by hematologists to screen for RBC membrane disorders. Therefore, the development of new diagnostic tools able to perform analysis at the scale of a single cell, over a statistically relevant population, would provide important complementary information. But these new diagnostic tools would have to be able to discriminate between different disorders causing a change in RBCs mechanical properties. We evaluated the mechanical response of artificially rigidified RBCs flowing through a microfluidic constriction. The geometry consists in a 50 μm wide channel with a succession of 14 tooth-like patterns, each composed of a 5 μm wide and 10 μm long constriction, associated with a 25 μm wide and 10 μm long enlargement. RBCs deformability was altered using two chemical treatments, known to affect RBCs membrane surface area and membrane deformability, lysolecithine (LPC) and diamide, respectively. Differences between samples were highlighted by the representation of the inverse of the shape recovery time (1/*τ_*r*_*), versus the extension at the exit of the constriction, *D*_*out*_. The results demonstrate that our approach is able to provide a direct signature of RBCs membrane composition and architecture, as it allows discriminating the effect of changes in RBCs membrane surface area from changes in RBCs membrane deformability. Finally, in order to evaluate the potential of our microsystem to detect pathological cells, we have performed preliminary experiments on patients with Hereditary Spherocytosis (HS) or Sickle Cell Anemia (SCA).

## Introduction

Red Blood Cells (RBCs) membrane possesses a unique structure responsible for their remarkable ability to deform to flow through the small capillaries of the microcirculation. RBCs have an elastic 2D mesh-like spectrin cytoskeleton anchored to the internal side of a lipid bilayer (Mohandas and Evans, 1994). The membrane of RBCs encloses a cytoplasm made of hemoglobin which viscosity is 10 mPa.s at 25°C. The membrane and the absence of nucleus in the internal media, plays a key role in the regulation of RBCs deformability.

Under normal conditions, the membrane deforms at constant surface area and exhibits a viscoelastic behavior. Once the cell surface area excess has been unfolded, further extension of the membrane is governed by the lipid bilayer, which tends to resist area expansion. The shear resistance of the membrane is directly related to the density of spectrin and thus, to the cytoskeleton molecular structure (Waught and Agre, 1988). RBCs have been extensively studied in the past 50 years in order to characterize mechanical phenotypes associated with both healthy and pathological states.

Various diseases such as malaria (Shelby et al., 2003; Suresh et al., 2005; Mauritz et al., 2010) diabetes (Buys et al., 2003) Sickle Cell Anemia (Ballas and Mohandas, 2004; Maciaszek and Lykotrafitis, 2011; Iragorri et al., 2018) (SCA) or Hereditary Spherocytosis (Waught and Agre, 1988) (HS) are associated with variation of RBCs deformability. Although conventional techniques allowing the quantification of cellular mechanical properties, (Suresh, 2007) such as atomic force microscopy (AFM), micropipette aspiration and optical tweezers are well-established, they present throughput too low (of the order of several tens of cells per day) to be envisaged as routine diagnostic tools. Currently only osmotic gradient ektacytometry (osmoscan), which consists in following the behavior of a suspension of RBCs sheared into a Couette system across an osmotic gradient, is used by hematologists to screen for RBC membrane disorders (Mohandas et al., 1980; Da Costa et al., 2013, 2016). However, up to now, they cannot provide any quantitative information such as parasitemia for malaria or distribution of cell populations which would provide valuable insights for SCA or HS (Dondorp et al., 1997).

Due to a match between cell sizes and typical dimensions accessible by microfabrication methods, microfluidic technologies propose attractive solutions for the study of cellular mechanics at the single cell level, while being compatible with high throughput (Antia et al., 2008; Hauck et al., 2010). Previous studies have demonstrated the use of various microfluidic geometries (Tomaiuolo, 2014) to detect alteration of RBCs deformability. Very different readouts have been used, such as deformation index, cell flowing velocity, transit time, relaxation time, etc. For example, Tsukada et al. (2001) have shown that the shape of cells flowing in micro-capillaries according to their velocity can be used to discriminate diabetic from healthy RBCs. Shelby et al. (2003) have shown that the ability of malaria infected RBCs to flow through microfluidic constrictions could be related to the stage of maturation of the parasite inside the host cells. Zheng et al. (2013) combined the flow of RBCs in a geometric constriction and impedance measurement to differentiate adult from neonatal RBCs. Guo et al. (2012) have measured the cortical tension associated with healthy, and *Plasmodium falciparum* infected RBCs, using funnel channels. Differences in velocities of RBCs flowing through geometrical constrictions have been used to discriminate healthy and diamide treated RBCs (Vilas Boas et al., 2018) or healthy and malaria infected RBCs (Bow et al., 2011). Faustino et al. (2014) took advantage of the strong extensional flow associated with a microchannel implementing a hyperbolic constriction, to differentiate RBCs from RBCs in contact with tumoral cells and healthy RBCs from RBCs of End-Stage Kidney Disease (ESKD) patients (Faustino et al., 2019). Tomaiuolo et al. (2011) have used the behavior of RBCs flowing through converging constrictions in order to evaluate cell membrane viscoelastic properties. Several papers have focused on the measurement of RBCs relaxation time (Tomaiuolo and Guido, 2011; Braunmüller et al., 2012; Prado et al., 2015) in microfluidics – i.e., the time necessary for the cell to recover its discocyte-like shape after cessation of the flow.

Although many research teams have demonstrated the ability to detect modification of molecular structure, (Forsyth et al., 2010; Hansen et al., 2011; Guo et al., 2012) to the best of our knowledge, none of them have tried to evaluate the specificity of their measurement by demonstrating their ability to discriminate between several diseases inducing an overall stiffening of the cells.

While many researches focused on the flow of RBCs through a microfluidic geometric constriction, we explored for the first time the maximum deformation of the cells being stretched by the sudden extension of the channel, in relation to its shape recovery time. We report the effect of two chemical treatments known to affect RBCs membrane surface area or membrane deformability on the dynamical behavior of RBCs flowing out a microfluidic constriction. We evaluated whether the response of the cells at the exit of the constriction was sensitive enough to discriminate between both effects (i.e., excess surface area and membrane elasticity). Finally, preliminary results highlighting the mechanical responses of RBCs in few patients with HS and SCA are discussed, thus addressing the specificity of our approach for potential diagnosis applications.

## Materials and Methods

### Blood Samples

#### Healthy Samples

Healthy blood samples were collected in EDTA tubes. Mechanically impaired RBCs were obtained by treating healthy RBCs with one of the following molecules: lysolecithin (LPC) or diamide (Clark et al., 1983). Before and after treatment, RBCs were washed twice with phosphate buffered saline (PBS) 1X (Biosolve chemicals BV, Netherlands) and then re-suspended at a concentration of 19 × 10^6^ RBCs/mL – corresponding to a hematocrit (Ht) of 0.17% – in PBS 1X. This dilution was necessary in order to avoid the flow of several cells simultaneously in the microchannel. Finally, after centrifugation, RBCs were re-suspended in dextran solutions (Sigma-Aldrich, Saint-Louis, MO, United States) (Dextran from *Leuconostoc* spp., *M*_*w*_ = 2 × 10^6^ g/mol was used) at 0.9 mg/mL of PBS 1X, to avoid RBCs sedimentation in the reservoir and guaranty the injection of a homogeneous concentration of cells during the time of an experiment while maintaining the low Ht condition. Dextran solutions were always filtered at 0.2 μm on the day of use. The use of dextran solutions also increased the hydrodynamic stress undergone by the cells in the channel. Viscosity, pH and osmolarity of the solutions were verified to be 31.5 mPa.s, 7.4 and 300 mOsmol, respectively.

#### Cell Surface and *S/V* Reduction (LPC Treatment)

Washed RBCs were incubated for 5 min at room temperature with LPC (Sigma-Aldrich, Saint-Louis, MO, United States) (LPC from egg yolk with *M*_*w*_ = 505 g/mol was used) at final concentrations ranging from 0 to 1.0 μmol/mL of cells, according to the protocol reported by Clark et al. (1983) LPC changes discocyte RBCs to type III echinocytes, induces membrane vesiculation and loss of membrane, resulting in a reduction of the surface to volume ratio (noted *S/V*).

#### Membrane Deformability Reduction (Diamide Treatment)

As previously described,30,31 washed RBCs were incubated with diamide (Sigma-Aldrich, Saint-Louis, MO, United-States) at final concentrations ranging from 0 to 1.0 mmol/L for 1 h at 37°C. Diamide induces the formation of disulfide bonds between spectrin proteins and increases the shear modulus of RBCs (Fischer et al., 1978; Safeukui et al., 2012).

#### Pathological Samples

Blood samples from 3 healthy individuals, 3 patients with HS and 2 SCA patients were collected in EDTA tubes. The protocol was approved by the “Hospices Civils de Lyon – CPP Est” Ethics Committee (L14-127).

### Device Fabrication

**FIGURE 1:**
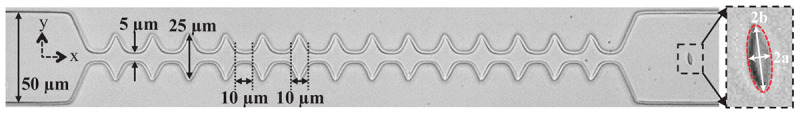
The geometry consists in a 50 μm wide channel implementing 14 tooth-like patterns. The height of the device is 10 μm. The different dimensions are reported on the pictures. The inset shows a close-up of a RBC which contour is fitted by an ellipse (dashed red line). The two axes of the ellipse along (2a) and perpendicular (2b) to the flow direction allows the calculation of a deformation index such as *D* = (2a–2b)/(2a + 2b).

Microfluidic channels in polydimethylsiloxane Sylgard 184 (PDMS) were manufactured using standard soft photolithographic techniques (Duffy et al., 2004) and sealed on glass via oxygen plasma treatment (Harrick Plasma, Ithaca, NY, United States). The geometry consisted in a 50 μm wide and 10 μm high channel, in which a succession of 15 tooth-like patterns have been implemented as illustrated in Figure 1. Each teeth-like pattern was composed of a 5 μm wide and 10 μm long constriction, associated with a 25 μm wide and 10 μm long enlargement. This width oscillation has been repeated over 290 μm and was chosen for its ability to significantly center the RBCs at the exit of the last constriction.

### Video-Microscopy

Polyethylene (PE 20) tubes (Harvard Apparatus, Holliston, MA, United States) connected the blood reservoirs to the inlet hole in the device and the outlet hole to the trash reservoir. RBC suspensions were injected in the microsystems by the flow control system MFCS^TM^-EZ (Fluigent, Paris, France) at a pressure of 200 mbar. Video-microscopic recordings of the cell behavior were performed with an inverted phase contrast microscope (Leica DMI 4000B, Germany) with a 40× magnification and a high speed camera (Mikrotron EoSens MC1362, Germany). The microscope was equipped with an environmental chamber (Ibidi, Martinsried, Germany) thus allowing the experiments to be done at 37°C.

### Image Analysis

Post processing of the movies was performed using a self-edited Matlab code to study cell dynamics and deformation. Briefly, on each image, after background subtraction, allowing to get rid of the microchannel walls, RBCs were automatically detected and cells contour was fitted with an elliptical shape. The position of the ellipse center of mass, the length of both axes along (*x*-direction) and perpendicular to (*y*-direction) the flow direction (2*a* and 2*b*, respectively, see inset Figure 1), were measured, allowing the calculation of the deformation index *D* defined as *D* = (2*a*−2*b*)/(2*a* + 2*b*). Currently, the routine takes a couple of minutes (2–5 min) per cell on a regular computer, because the process is not fully automatized yet, which account for the quite low number of cells investigated (∼60 cells/sample). However, this step could be optimized in the future to reach nearly real-time analysis as reported by Deng and Chung (2016) on similar analysis with a throughput of 2000 cells/s.

For each condition, roughly 50 cells were analyzed. Results are presented as box-and-whisker plots. A non-parametric ANOVA test for independent measurements was used to compare the different readouts before and after chemical treatments. *Post hoc* comparisons were performed using Fisher’s Least Squares Difference method. A Multivariate Analysis of Variance (MANOVA) test was used to compare the different pathological samples (HS, SCA, and control samples). The significance level was defined as *p* < 0.05.

### Ektacytometry

Osmoscan experiments have also been performed on the LoRRca MaxSis^®^ device (RR Mechatronics, Hoorn, Netherlands) to confirm the effects of the different molecules tested on RBCs, as well as to verity the presence of the specific osmoscan signatures already described in HS and SCA patients. Osmoscan consisted in the measurements of a RBC deformability or elongation index (*EI*) under a defined shear stress (30 Pa), at increasing osmolality from 90 to 600 mOsm/kg and at 37°C, as recommended (Nemeth et al., 2015; Da Costa et al., 2016; Zaninoni et al., 2018). Buffer viscosity was 30.4 mPa.s. Several parameters were determined: *O*_*min*_ (i.e., the osmolality at which RBC deformability value reaches a minimum in the hypotonic region of the curve), *EI*_*max*_ (i.e., the highest RBC deformability) and *O*_*hyper*_ (also called *O’*), which corresponds to the osmolality at half of the *EI*_*max*_ on the hypertonic region of the curve (Zheng et al., 2013). *O*_*min*_ reflects the osmotic fragility and the surface-to-volume ratio, *EI*_*max*_ depends on the membrane deformability and RBC surface area, and *O*_*hyper*_ reflects Mean Cellular Hemoglobin Concentration (MCHC) and Mean Cell Volume (MCV) and is therefore highly dependent on the hydration status of the cells (Clark et al., 1983; Da Costa et al., 2013).

## Results and Discussion

### Typical Behavior of Healthy RBCs

**FIGURE 2:**
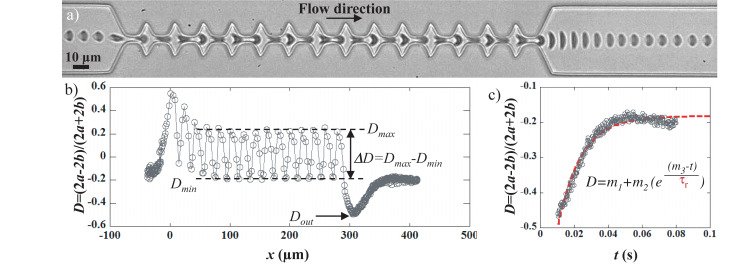
**(a)** sequence of deformation of a healthy RBC flowing in the geometry at 200 mbar and **(b)** the associated variation of the deformation index D [*D* = (2a–2b)/(2a + 2b)] as a function of the position of the cell center of mass. The origin of the graph has been arbitrarily chosen at the entrance of the first narrowing. The amplitude of deformation *ΔD* is calculated as *ΔD* = *D*_*max*_–*D*_*min*_, where *D*_*max*_ and *D*_*min*_ are the mean of D in the constrictions and the enlargements, respectively. The minimum of the curve corresponds to the large cell elongation at the exit of the last constriction, noted *D*_*out*_. **(c)** representation of *D* versus time of the previous graph, where only the time window of the cell recovery is reported. The exit of the last narrowing was set to be *t* = 0. The dashed red line is an exponential fit allowing the determination of the recovery time *τ_*r*_* according to the following equation *D* = *m*_1_ + *m*_2_ exp((*m*_3_–*t*)/*τ_*r*_*), where *m*_1_, *m*_2_, and *m*_3_ are constant to be adjusted.

Video-microscopic recordings of RBCs flowing in the microfluidic channel allowed the visualization of the cell deformation – quantified using the deformation index *D* – as it travels through the device. Figure 2 presents the typical behavior of a RBC flowing in the geometry of interest. The sequence of deformation associated with the flow of a healthy RBC in the geometry is reported in Figure 2a and the associated deformation index *D* is presented in Figure 2b. The cell flowing in the 50 μm wide channel presents a slipper-like shape, typical of RBC in confined flow (*D* ≈−0.2). As it approached the first constriction, the cells got compressed which is traduced by its elongation along the flow direction (*x*-axis). Indeed, *D* rose until reaching a maximum value around *x* = 0. Then, it underwent a stretching along the *y*-axis (i.e., perpendicular to the flow direction) when entering in the enlargement, before being compressed again by the next constriction. Accordingly, *D* dropped until reaching a local minimum (for *x* = 10 μm), before increasing again at *x* = 20 μm. This cycle of compressing/stretching is repeated due to the tooth-like patterns as illustrated by the oscillations of *D* versus *x*. The cyclic deformation of the cell within the geometry can be characterized through the measurement of the amplitude of deformation *ΔD* defined as *ΔD* = *D_*max*_−D_*min*_*, where *D*_*max*_ and *D*_*min*_ are the mean of *D* in the constrictions and the enlargements, respectively, as illustrated in Figure 2b. Finally, as it exited the last narrowing, around *x* = 290 μm, RBC got strongly stretched perpendicular to the flow direction, which is traduced by the sudden drop of *D*. After reaching a minimum value noted D_out_ around *x* = 310 μm, *D* returns slowly to its initial value and reaches a plateau corresponding to the steady slipper-like shape.

The variation of *D* between the last deformed state at the exit, *D*_*out*_, and the final steady shape, corresponding to the plateau value, is represented versus time in Figure 2c. The experimental data were fitted using an exponential growth, hence allowing the extraction of the shape recovery time *τ_*r*_* defined as the time necessary for the cell to return to a stationary shape after exiting the last constriction, while being still under hydrodynamic stress. We have measured the shape recovery time of healthy RBCs while varying different viscous stress applied, i.e., hydrodynamic parameters such as buffer viscosity and cell speed (Supplementary Figure SI.2B). Because RBC steady shape is reached under hydrodynamic stress rather than at rest, the recovery time of RBCs does not directly correspond to their relaxation time. Indeed the relaxation time is defined as the time necessary for the cell to adopt the resting « discocyte-like » shape after total cessation of any stress. As already reported in the literature, the relaxation time τ depends only on the intrinsic mechanical properties of the RBC membrane, i.e., τ = η_m_/μ, where η_m_ is the 2D membrane viscosity and μ the membrane shear modulus. According to the literature, τ has been measured using different techniques to be in the range 100–300 ms (Hochmuth et al., 1979; Mohandas et al., 1980; Braunmüller et al., 2012). Indeed, in specific experimental conditions, recovery times were measured at τ_r_ = 129 ms for η_out_ = 1.3 mPa.s and V_cell_ = 170 μm/s, which is in good agreement with values previously reported in the literature for the relaxation time (Hochmuth et al., 1979; Mohandas et al., 1980; Evans, 1989; Tomaiuolo and Guido, 2011; Braunmüller et al., 2012). But they can also reach values as low as τ_r_ = 4 ms for η_out_ = 20.3 mPa.s and V_cell_ = 1500 μm/s (Amirouche et al., 2020). We could explain these results in terms of coupling between the cell and the flow. This assumption would explain why at low hydrodynamic stress – where the cell properties dominate the recovery process and where the hydrodynamics can be neglected - we can assume being in an almost static configuration, the recovery time tend toward relaxation time values. In a previous study, we reported that at fixed hydrodynamic stress, shape recovery times of RBCs can be used to discriminate between healthy and mechanically impaired RBCs (Amirouche et al., 2017). In the present paper, we aim at demonstrating that our approach is sensitive enough to discriminate between different membrane modifications.

The repetition of the teeth-like pattern tends to focus cells within the microchannel (see Supplementary Figure SI.1 in Supplementary Information), hence ensuring a symmetrical deformation at the exit of the last narrowing. This prevents them from rotating and imposes that all cells are exposed to the same hydrodynamic stress, all RBCs being aligned on the same flow line. Despite this advantage, the repetition of the constriction could also be a drawback. Indeed, it has been reported in literature (Lee et al., 2004; Simmonds and Meiselman, 2016; Horobin et al., 2017; Qiang et al., 2019) that the application of a stress too high or for a too long period of time can induce a mechanical fatigue of the cells. For example, Simmonds et al. report that RBCs present impaired deformability when exposed to physiological levels of shear stress (Simmonds and Meiselman, 2016) (above 40 Pa) for 1–64 s. Other studies report that cyclic mechanical solicitations of RBCs lead to significantly greater loss of membrane deformability, compared to continuous deformation under the same maximum load and duration (Lee et al., 2004). Therefore, we have verified that the repetition of the restriction did not impact the behavior of RBCs exiting the geometry. As presented in Supplementary Figure SI.2, the shape recovery time of healthy RBCs was identical after exiting a single 10 μm long constriction and the 15 repetitions of a 10 μm long constriction, hence demonstrating that no fatigue was detected in our experiments.

### Chemically Altered RBCs

**FIGURE 3:**
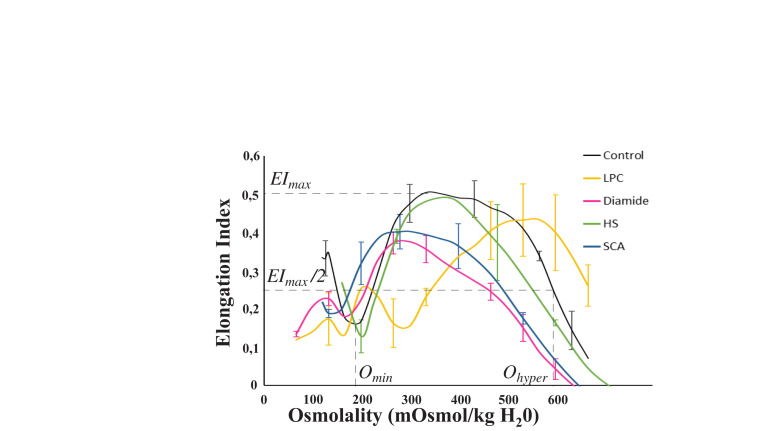
Schematic representation of Osmoscan experiments. Several parameters can be determined on this curve: *O*_*min*_ (i.e., the osmolality at which RBC deformability value reaches a minimum in the hypotonic region of the curve), *EI*_*max*_ (i.e., the highest RBC deformability) and *O*_*hyper*_ which corresponds to the osmolality at half of the *EI*_*max*_ on the hypertonic region of the curve. LPC treated RBCs at 1.0 μM show an increase in *O*_*min*_ and *O*_*hyper*_ and a decrease in *EI*_*max*_. Diamide treated RBCs at 0.5 mM show a decrease in *EI*_*max*_ and *O*_*hyper*_. These HS patients have a decreased *EI*_*max*_ and *O*_*hyper*_ compared to control. *O*_*min*_, *O*_*hyper*_ and *EI*_*max*_ are decreased in these SCA patients compared to control.

**FIGURE 4:**
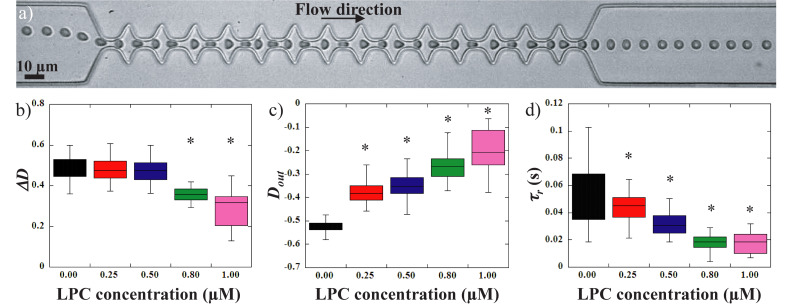
**(a)** sequence of deformation of a 1 μM LPC treated RBC flowing in the geometry. Evolution of **(b)** the amplitude of deformation *ΔD*, **(c)** the stretching at the exit *D*_*out*_ and **(d)** the recovery time *τ_*r*_*, versus LPC concentration. *Different from control sample (0 μM), *p* < 0.05.

**FIGURE 5:**
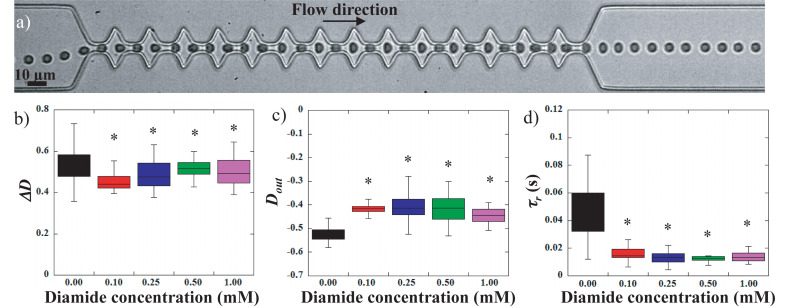
**(a)** sequence of deformation of a 1 mM diamide treated RBC flowing in the geometry. Evolution of **(b)** the amplitude of deformation *ΔD*, **(c)** the stretching at the exit *D*_*out*_, and **(d)** *τ_*r*_*, the recovery time versus diamide concentration. *Different from control sample (0 mM), *p* < 0.05.

Two chemical treatments were performed on healthy RBCs in order to affect RBC deformability. For each sample, osmotic gradient ektacytometry was performed (some results are shown on Figure 3) and the mechanical responses of RBCs flowing in the microfluidic system were evaluated (Figure 4 for LPC treated RBCs, and Figure 5 for diamide treated RBCs). LPC is known to cause a reduction in the surface area of RBCs (Mohandas et al., 1980; Clark et al., 1983). Osmotic gradient ektacytometry experiments performed on LPC treated RBCs at 1.0 μM (Figure 3) showed a significant rise in Omin and Ohyper, and a decrease in EImax. These findings confirm the effects of LPC on cell sphericity (decrease of surface-to-volume ratio). The increase in Ohyper could be explained by the slight increase in cell volume upon LPC treatment (MCV were measured to be 87.8 and 103.4 fL for the control and 1.0 μM LPC treated sample, respectively) as already reported (Safeukui et al., 2012). Figure 4 reports the effect of LPC at various concentrations (from 0.25 to 1 μM) on the RBCs mechanical response while flowing in the geometry. Figure 4a shows a typical sequence of deformation of a LPC-treated cell at 1 μM.

Lysolecithine treated RBCs behaved qualitatively similarly to healthy RBCs. They got compressed and stretched according to the width of the channel, and got elongated by the extensional flow at the exit before recovering a stationary shape. However, a reduction of the amount of deformation experienced by the cells can be visually detected on the picture while undergoing the same amount of stress. These observations are confirmed by the measurements of the amplitude of deformation, *ΔD* versus LPC concentration upon treatments (Figure 4b). LPC treatment at a concentration up to 0.5 μM had no significant impact on the amplitude of deformation of RBCs compared to healthy RBCs. However, treatment with higher concentrations led to a significant reduction of *ΔD* (Figure 4b). Figures 3d, 4c show the evolution of the elongation at the exit, *D*_*out*_, and the recovery time, *τ_*r*_*, respectively. The stretching at the exit, *D*_*out*_, and the recovery time, *τ_*r*_* were gradually decreased upon increasing LPC concentration; hence showing that RBCs deformability drops gradually with the concentration of LPC.

Previous works used diamide to rigidify the RBCs membrane (Mohandas et al., 1980; Forsyth et al., 2010; Prado et al., 2015). Indeed, diamide treatment (Figure 3) decreased *EI*_*max*_ and *O*_*hyper*_. The increase in diamide concentration caused asymmetry in the hump of the osmotic gradient ektacytometry curve with a greater reduction in *EI* on the hypertonic part of the curve than on the hypotonic side. These findings confirmed the effects of diamide on membrane deformability, i.e., an increased shear modulus caused by the cross-linking between spectrins (Mohandas et al., 1980). Figure 5 illustrates the effect of diamide treatment with concentrations ranging from 0.1 to 1.0 mM. Figure 5a presents the typical sequence of deformation of a diamide treated RBC at 1 mM. As for LPC treatments, RBCs deformability was clearly reduced when the cell was incubated with diamide, although the qualitative behavior of the cells was similar to that of healthy RBCs. Diamide treated cells showed a slight, yet statistically significant, drop in the amplitude of deformation *ΔD*, as illustrated in Figure 5b. Nevertheless, upon incubation with diamide, elongation at the exit *D*_*out*_, of treated RBCs was reduced (Figure 5c), although the measurements were not able to make a distinction between the different diamide concentrations. Diamide treatment also impacted the recovery time *τ_*r*_* of RBCs as highlighted by the decrease from 0.048 s for healthy RBCs to roughly 0.013 s for diamide treated cells (Figure 5d).

From the results presented above, it seems that *ΔD* is not sensitive enough to clearly detect modifications of the cells mechanical properties. This may be explained by the fact that this parameter is highly impacted by the various off-centered initial positions of the cells when entering the zone of interest.

**FIGURE 6:**
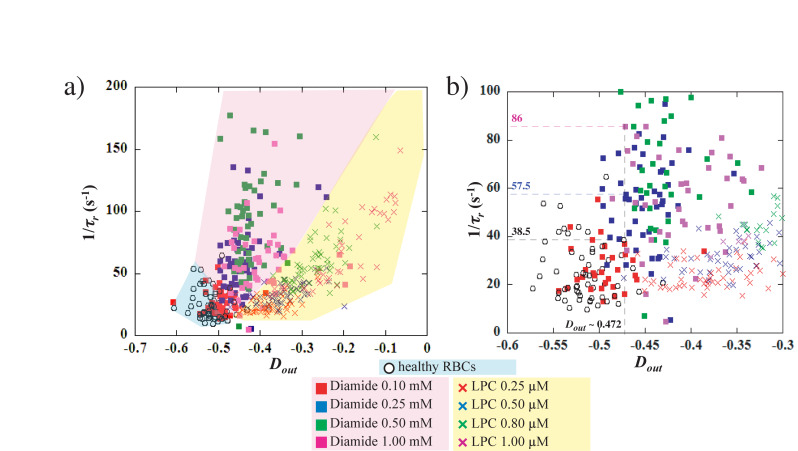
**(a)** evolution of 1/*τ_*r*_* versus *D*_*out*_ for healthy, Diamide and LPC treated RBCs. **(b)** close-up of the graph in **(a)**. For a given value of the stretching at the exit, *D*_*out*_ = –0.472, healthy RBC presents 1/*τ_*r*_* = 33.5 s^–1^, 0.25 mM diamide treated cell has 1/*τ_*r*_* = 57.5 s^–1^ and 1 mM diamide treated cell has 1/*τ_*r*_* = 86 s^–1^.

However, the measurements of the maximum elongation at the exit *D*_*out*_ and the RBCs recovery time τ_r_ can be used to discriminate healthy from chemically treated RBCs. But whether these readouts can differentiate between the different chemical concentrations is unclear. In the case of a visco-elastic object such as a RBC, the relaxation time, and therefore the recovery time, is linked to the deformed state. Indeed, *τ_*r*_* decreases as *D*_*out*_ decreases, i.e., it takes less time to recover from a less elongated shape than from a more elongated one. Figure 6 represents 1/*τ_*r*_* versus *D*_*out*_, for the different concentrations of diamide and LPC as well as the healthy samples. It can be clearly observed on the figure that, while healthy RBCs mechanical signature is limited to low 1/*τ_*r*_* (<50 s^–1^) values and strong elongation (*D*_*out*_ < −0.5), chemically rigidified RBCs present lower elongation at the exit associated with shorter recovery time. We can see on Figure 6b, that for a given extension at the exit D_out_∼−0.472, 3 RBCs present three different values of recovery times according to diamide concentration: 1/τ_r_ = 38.5 s^–1^ for the healthy cell (τ_r_ ∼26 ms), 1/τ_r_ = 57.5 s^–1^ for the RBC treated with diamide at 0.25 mM (τ_r_∼17 ms) and 1/τ_r_ = 86 s^–1^ for 1 mM diamide treated RBC (τ_r_∼12 ms). These observations suggest that cells treated with diamide (at a concentration above 0.1 mM) exhibit a recovery time smaller than that of healthy ones for an equivalent extension at the exit D_out_. This representation allows to discriminate easily the chemically rigidified RBCs from the healthy ones (blue zone). Moreover, it is possible to differentiate the two treatments as all the data points associated with LPC treated cells are located in a region (yellow zone) of the graph while diamide treated RBCs are located in another region (pink zone). Although both treatments lead to an increase in the overall cell rigidity, which is traduced by a lower *D*_*out*_ and a shorter *τ_*r*_*, we can distinguish the effect of increased membrane stiffness from the effect of excess surface area.

The results obtained with chemically treated cells highlight that our microsystem is not only able to quantify the RBCs ability to deform, but also to provide a direct signature of the composition and architecture of their membrane. Indeed, we suspect that our microfluidic approach could be useful to differentiate and diagnose different kind of RBC disorders according to the origin of the affection (RBC membrane disorders, changes in surface-to-volume ratio, etc…).

### Pathological RBCs

**FIGURE 7:**
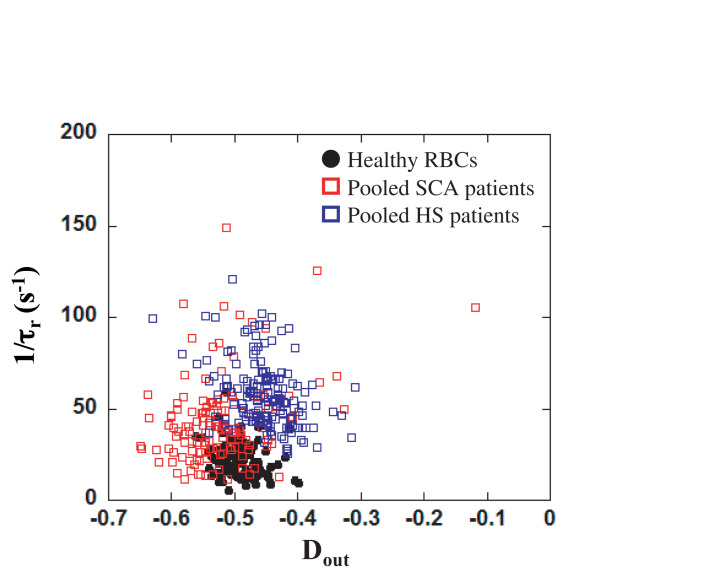
*D*_*out*_ versus 1/*τ_*r*_* for healthy RBCs (solid black circles), pooled SCA patients (open red squares) and pooled HS patients (open blue squares). A MANOVA statistical analysis revealed that the RBCs distribution was significantly different between the three groups (*p* < 0.05 between HS and healthy, *p* < 0.05 between SCA and healthy, and *p* < 0.05 between HS and SCA).

In order to evaluate the potential of our microsystem to detect pathological cells, we have studied the mechanical response of HS and SCA RBCs flowing out of our tooth-like pattern. Osmoscan experiments (Figure 3) showed a typical signature obtained for HS and SCA RBCs by ektacytometry. Figure 7 reports the measurements of 1/*τ_*r*_* as a function of *D*_*out*_ for three HS patients, two SCA patients and one healthy donor. In order to help decipher if the two pathological samples are not only different from the healthy sample but also between each other, we decided to represent all pathological data on the same graph. SCA data correspond to the two different patients pooled together. In the same way, the points corresponding to the HS RBCs, were obtained by pooling the three HS patients. As illustrated on the Figure, RBCs from HS patients showed an increase of 1/*τ_*r*_* compared to healthy RBCs, while RBCs from SCA patients had both a decrease in *D*_*out*_ and a slight increase in 1/*τ_*r*_*. Such increase in 1/*τ_*r*_* is expected because these diseases are known to increase cell stiffness (Nakashima and Beutler, 1979; Waught and Agre, 1988; Da Costa et al., 2013).

A MANOVA analysis showed that the distribution of RBCs according to *D*_*out*_ and 1/*τ_*r*_* was significantly different between HS, SCA and control individuals (*p* < 0.05). The morphologic signature of HS is the presence of microspherocytes (smaller spherical RBCs), which is caused by loss of RBC membrane surface area, leading to an abnormal osmotic fragility. Although one would have expected to find similar response with HS patients than with LPC treated cells, both being associated with a loss of membrane surface area, other modifications of HS RBCs may explain the difference between the LPC and HS microfluidic signatures. For instance, HS blood also contains dense RBCs (usually more than 4% of RBCs with MCHC > 41 g/dL) which may impact on the microfluidic behavior of the cells (Mohandas et al., 1980; Da Costa et al., 2016). Finally as usual, the inter-cells variability within a blood sample was higher in SCA than in HS. This discrepancy could be attributed to the presence of different sub-populations of more or less dense cells in SCA, suggesting the possibility to retrieve information about the composition of the different sub-populations. Although only few patients were tested, our preliminary results seem to highlight that our microfluidic device could be sensitive enough to make a distinction between healthy and pathological RBCs. Nevertheless, HS patients included in this study were issued from the same family, i.e., presenting the same genetic molecular modifications, and it remains unknown whether our system is able to discriminate RBCs from HS patients with different molecular modifications (ankyrin, α–spectrin, β–spectrin …). However, in this particular HS family, with the same red cell membrane molecular defect, we reproduced consistently in the three affected relatives the same microfluidic signature.

## Conclusion

Differences between healthy and chemically treated cells were highlighted by the measurement of both the extension at the exit, *D*_*out*_, and the shape recovery time *τ_*r*_*. Although the measures were not able to distinguish between the different chemical concentrations, the representation of 1/*τ_*r*_* versus D_out_ membrane surface area from membrane elasticity. It is able to provide a direct signature of RBCs membrane composition and architecture.

Our preliminary results highlight that the mechanical responses of healthy and pathological RBCs are not only different but also suggest that the discrimination of both diseases could be possible, although more experiments using samples from various RBC disorders are needed. To our knowledge, this is the first attempt to evaluate the specificity of a passive microfluidic approach to perform diagnosis based on the alteration of RBC deformability, although more experiments are necessary to prove it. In addition to is diagnostic potential, further studies including large cohort of patients would be necessary to evaluate the clinical usefulness of our microfluidic approach for various RBC disorders. The single cell approach, while analyzing a statistically relevant population, could also provide complementary information (quantification of the heterogeneity of the cell population) that might be of great importance for the diagnosis and prognosis.

## Data Availability Statement

The datasets generated for this study are available on request to the corresponding author.

## Ethics Statement

The studies involving human participants were reviewed and approved by the “Hospices Civils de Lyon – CPP Est” Ethics Committee (L14-127). The patients/participants provided their written informed consent to participate in this study.

## Author Contributions

MF, CR, PJ, and PC have designed the research. MF and AB have performed the microfluidic experiments. CR and AB have performed the ektacytometry assays. LD and AG included the patients. MF, CR, and PC have analyzed the results. MF has written the main manuscript text and prepared the figures. MF, CR, LD, PJ, AG, and PC have reviewed and approved the final version of the manuscript.

## Conflict of Interest

The authors declare that the research was conducted in the absence of any commercial or financial relationships that could be construed as a potential conflict of interest.

## Author’s Note

‡ The repetition of the tooth-like pattern tends to focus cells at the exit. Such behavior has also been observed at the exit of very long straight constrictions, however, it would need a fairly longer geometry to obtain the same effect.
