# Supplementary material for: Mechanical Signature of Red Blood Cells Flowing Out of a Microfluidic Constriction Is Impacted by Membrane Elasticity, Cell Surface-to-Volume Ratio and Diseases
Source: Front Physiol. 2020 Jun 12;11:576. doi: 10.3389/fphys.2020.00576 (PMC7303906; doi:10.3389/fphys.2020.00576)
Supplement: Supplementary file 1 [file Data_Sheet_1.PDF]

Supplementary information

Mechanical signature of Red Blood Cells flowing out of a microfluidic constriction is impacted by membrane elasticity, cell surface-to-volume ratio and diseases

Magalie Faivre,*[a] Céline Renoux,[b,c,d] Amel Bessaa,[b,c] Lydie Da Costa,[c,e,f,g] Philippe Joly,[b,c,d] Alexandra Gauthier[h] and Philippe Connes[b,c,i]

Centering effect of the specific channel geometry.

RBCs flowing through a single 10 µm long and 5 µm wide constriction, would experience a symmetrical trajectory, thus exiting the geometric restriction on the same flow line that they followed when entering. This would result in a large variety of RBC behaviors while exiting the constriction, most of them being associated with a non-symmetrical stretching at the exit, which would make data difficult to interpret. One huge advantage of the specific geometry used in this paper, i.e. the width oscillation of the channel or the repetition of the geometric restriction, associated with widenings, is illustrated on Figure SI.1. Indeed, it can be seen that whatever the initial position of the cells within the channel, the RBCs tend to exit centered, hence ensuring a symmetrical stretching at the exit and a symmetric recovery of stationary shape.

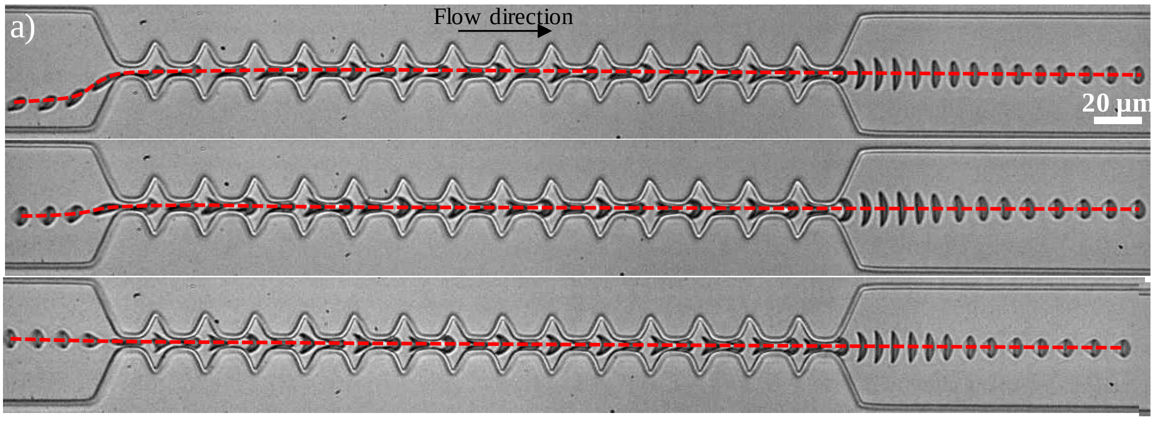

Figure SI.1: Sequence of deformations of 3 healthy RBCs entering at 3 different positions in the channel: RBCs center of mass is located a) 10 µm away from the lower wall, b) 20 µm away and c) 26 µm away, indicating a cell roughly centered relatively to the channel width (50 µm). The red dashed lines highlight the trajectory of the RBCs center of mass.

It could be noted that the same effect would be obtained with a single constriction, however, the narrowing would have to be much longer in order to have the same effect.

Effect of stress history.

We have verified that the repetition of the 10 µm long constriction did not impact the behavior of healthy cells. To do so, the recovery time of healthy RBCs were measured both after a single 10 µm long geometric restriction and at the exit of the 15 repetitions of the restriction. This study has been

conducted in channels with a main width of 15µm as illustrated in the inset of Figure SI.2a. In the Figure SI.2a, we present the recovery curves of two healthy RBCs flowing in both geometries. In order to facilitate the comparison and the interpretation of the data. The time has been arbitrarilly set to be the exit of the last constriction. We can see that the two recovery curves superimpose perfectly hence highlighting that there is no influence of the number of restrictions on the behavior of healthy RBCs exiting a 5 µm wide and 10 µm long constriction, i.e. D_{out} and τ_r. We also measured the shape recovery time of healthy RBCs in both geometries for different cell speeds. Figure SI.2b reports the inverse of recovery time, $1/\tau_r$ as a function of the cell speed. Cell speed - which is measured in the 15 µm wide section of the channel, far away after the exit of the restriction - is varied through the applied pressure at the entry of the microfluidic chip. Due to a difference in hydrodynamic resistance of both geometries, the same applied pressure results in different cell velocities. We showed that $1/\tau_r$ varies linearly with the cell speed. We verified that cells flowing in both geometries respond the same way as all the data collapse on the same linear regression, underlying that at a given cell speed, healthy cells present the same recovery time. Therefore we can conclude from thoses results that no irreversible deformations in the RBC membrane could be detected with our approach.

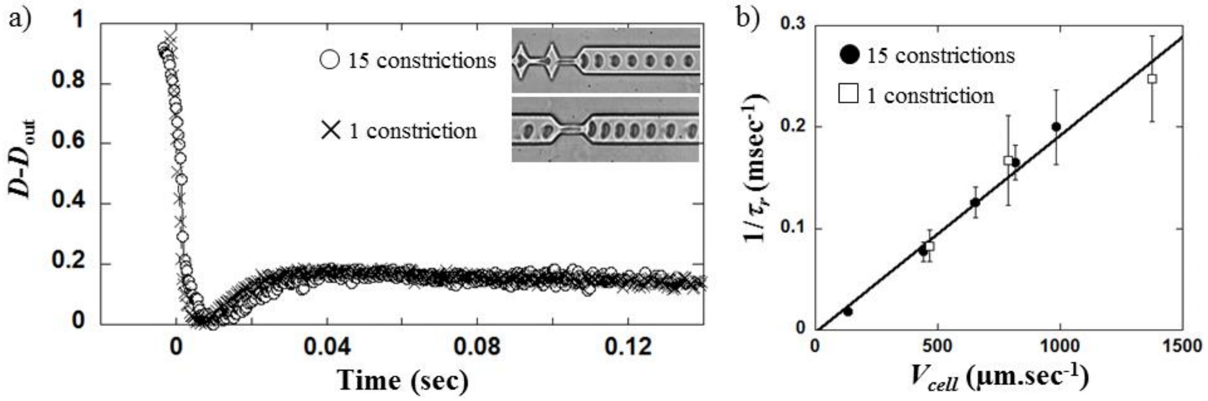

Figure SI.2 : a) Deformation index D versus time for two hRBCs flowing in a 15 µm wide channel implementing either a single 10 µm long and 5 µm wide constriction or 15 repetitions of a 10 µm long constriction of same width. The origine of the time has been arbitrarilly set to be the exit of the last constriction. V_{cell} = 500 µm/s and η_{out}=31.5 mPa.s. The deformation index has been normalized by the extension at the exit such as both cells present the same D_{out}. b) Evolution of $1/\tau_r$ versus cell velocity at the exit for both geometries for η_{out}=31.5 mPa.s.
